# Obesity-Related Discourse on Facebook and Instagram Throughout the COVID-19 Pandemic: Comparative Longitudinal Evaluation

**DOI:** 10.2196/40005

**Published:** 2023-05-16

**Authors:** Catherine Pollack, Diane Gilbert-Diamond, Tracy Onega, Soroush Vosoughi, A James O'Malley, Jennifer A Emond

**Affiliations:** 1 Department of Biomedical Data Science Geisel School of Medicine at Dartmouth Lebanon, NH United States; 2 Department of Epidemiology Geisel School of Medicine at Dartmouth Hanover, NH United States; 3 Department of Pediatrics Geisel School of Medicine at Dartmouth Hanover, NH United States; 4 Department of Medicine Geisel School of Medicine at Dartmouth Hanover, NH United States; 5 Department of Population Health Sciences Huntsman Cancer Institute University of Utah Salt Lake City, UT United States; 6 Department of Computer Science Dartmouth College Hanover, NH United States; 7 The Dartmouth Institute for Health Policy and Clinical Practice Hanover, NH United States

**Keywords:** obesity, Facebook, Instagram, COVID-19, social media, news, infodemiology, public health, online health information

## Abstract

**Background:**

COVID-19 severity is amplified among individuals with obesity, which may have influenced mainstream media coverage of the disease by both improving understanding of the condition and increasing weight-related stigma.

**Objective:**

We aimed to measure obesity-related conversations on Facebook and Instagram around key dates during the first year of the COVID-19 pandemic.

**Methods:**

Public Facebook and Instagram posts were extracted for 29-day windows in 2020 around January 28 (the first US COVID-19 case), March 11 (when COVID-19 was declared a global pandemic), May 19 (when obesity and COVID-19 were linked in mainstream media), and October 2 (when former US president Trump contracted COVID-19 and obesity was mentioned most frequently in the mainstream media). Trends in daily posts and corresponding interactions were evaluated using interrupted time series. The 10 most frequent obesity-related topics on each platform were also examined.

**Results:**

On Facebook, there was a temporary increase in 2020 in obesity-related posts and interactions on May 19 (posts +405, 95% CI 166 to 645; interactions +294,930, 95% CI 125,986 to 463,874) and October 2 (posts +639, 95% CI 359 to 883; interactions +182,814, 95% CI 160,524 to 205,105). On Instagram, there were temporary increases in 2020 only in interactions on May 19 (+226,017, 95% CI 107,323 to 344,708) and October 2 (+156,974, 95% CI 89,757 to 224,192). Similar trends were not observed in controls. Five of the most frequent topics overlapped (COVID-19, bariatric surgery, weight loss stories, pediatric obesity, and sleep); additional topics specific to each platform included diet fads, food groups, and clickbait.

**Conclusions:**

Social media conversations surged in response to obesity-related public health news. Conversations contained both clinical and commercial content of possibly dubious accuracy. Our findings support the idea that major public health announcements may coincide with the spread of health-related content (truthful or otherwise) on social media.

## Introduction

The SARS-CoV-2 virus and COVID-19 pandemic have fundamentally changed society. The first US case was reported on January 20, 2020, and the World Health Organization (WHO) declared COVID-19 a global pandemic on March 11. COVID-19 is an infectious respiratory disease associated with a range of symptoms, and severity may be amplified in individuals with chronic, preexisting conditions such as obesity. This link was first reported in the spring of 2020, and studies have estimated that obesity may increase the risk of hospitalization due to COVID-19 between 7% and 33% and death by 8% to 61% [1,2]. Patients with severe obesity are particularly susceptible; for example, studies have shown that these patients may have 7.36 higher odds of a need for invasive mechanical ventilation compared to normal-weight patients [3].

US mainstream media coverage of the association between COVID-19 severity and obesity peaked in October 2020, when then US president Trump contracted COVID-19 [4,5]. Given that the prevalence of obesity in the US now exceeds 40%, it is imperative to understand how discourse about the disease evolved both temporally and topically throughout the early stages of the pandemic [6]. Such knowledge can further elucidate how major events impact the public dialogue surrounding a chronic disease. On one hand, increased attention to obesity by the public may further understanding of obesity prevention and treatment; on the other hand, repeated negative portrayals of the disease, especially in the mainstream media, may amplify weight-related stigma [7].

Social media enables the documentation of heterogeneous opinions in near real time, making it an attractive avenue to assess shifts in opinion in response to major events. In particular, Facebook and Instagram are two popular social media platforms that were accessed by 70% and 59% of Americans on a daily basis in 2021, respectively [8]. The frequent use of these platforms by the public makes an evaluation of their content especially salient. While previous work on obesity discourse on these platforms during the pandemic has evaluated a small fraction of content, there has yet to be a comprehensive analysis of a large sample of public-facing content [9-11]. Reviewing a wide range of content on both Facebook and Instagram can further elucidate the interplay between mainstream and social media dialogue in the context of chronic diseases. Thus, the purpose of this study was to explore temporal and topical variations in obesity-related content on Facebook and Instagram throughout the first year of the pandemic to better contextualize the interplay between news media and public discourse as related to COVID-19 and obesity.

## Methods

### Overview

This study included temporal and topical analyses to characterize how obesity-related content evolved on Facebook and Instagram surrounding 4 major events related to COVID-19 in 2020. Two dates were selected given their relevance to the broader pandemic: January 20, the date of the first US case, and March 11, the date when the WHO declared COVID-19 a global pandemic. Two other dates were directly related to obesity: May 19, the approximate date that obesity and COVID-19 were linked, and October 2, the date when then US President Trump contracted COVID-19 and obesity was most discussed in the news media, according to data from Media Cloud, an open-source content aggregation tool [12]. Temporal analysis evaluated changes in the number of obesity-related posts and interactions on each platform around those dates. Topic modeling evaluated the 10 most frequent obesity-related themes on each platform, excluding content related to pet obesity.

### Data Collection

Facebook and Instagram posts were collected from CrowdTangle, a public insights tool owned and operated by Facebook [13]. CrowdTangle’s Facebook data encompassed public pages with over 25,000 likes or followers, public groups with over 95,000 members, US-based public groups with over 2000 members, and verified profiles (ie, user profiles that confirm the “authentic presence” of well-known public figures) [14]. CrowdTangle’s Instagram data encompassed public profiles with over 50,000 followers and verified profiles [15]. All content in English between January 6, 2020, and October 16, 2020, that contained the words “obese” or “obesity” was extracted. Health (“headache” or “migraine”) and nonhealth (“clarinet”) control data were extracted for the same period. Keywords for controls were chosen based on their perceived independence from obesity and for posting frequency that was within a degree of magnitude of the obesity data. For all Facebook data, posts made on animal-specific pages were removed; this kind of information was not available for Instagram data. On Facebook, interactions were defined as any kind of post reaction (such as likes or wows), comments, and shares. On Instagram, interactions were defined as likes, comments, and shares. When available, data also included metadata for the page, group, or profile on which the post was made, such as the category of page or group (eg, new organization, hospital).

### Temporal Modeling

Interrupted time series analysis was performed for each date using autoregressive integrated moving average (ARIMA) models. This method was chosen given its ability to control for highly cyclical and serially correlated data prior to each date and model complex postevent effects using one or a combination of transfer functions, including “pulse,” “step,” and “ramp” effects [16]. A pulse effect is characterized as an instantaneous increase on the day of the event followed by an immediate return to pre-event levels, a step effect is characterized as an instantaneous increase on the day of the event that is sustained after the event, and a ramp effect is a slope characterizing a differential rate of change in the outcome after the event [16]. All combinations of transfer functions were evaluated in separate models on the obesity data for a 29-day window around each date (ie, 14 days on either side of the event and the event itself). A 2-week postevent period was chosen to ensure that the impact of the event was captured. A shorter time window might not have captured the full extent of the event’s effect, while a longer window might have increased the likelihood of including a confounding event that would have precluded the ability to establish an association between the event of interest and the change in behavior. The same length of time was chosen in the pre-event period for symmetry.

Each model was developed using the *auto.arima* function in the “forecast” package in R to identify *p*, *d*, and *q* parameters [17]. Here, *p* represents the number of autoregressive lags (ie, how many past values of the outcome are needed to predict the current value), *d* represents the degree of nonseasonal differences to reach stationarity (with “stationarity” defined as a mean and variance independent of time), and *q* represents the number of lagged errors required to predict the outcome (ie, the number of lags in the moving average component of the model). The transfer functions in the model with the lowest sample-corrected Akaike information criterion (AICc) were chosen for each date. Parameter selection for *p*, *d*, and *q* was repeated for control models using the best transfer function set from the obesity model. A sensitivity analysis was run on control models using the same *p* and *q* parameters as the obesity model—in all cases, AICc values were higher, so only results from the model with data-specific *p* and *q* values are presented (Multimedia Appendix 1 includes both).

### Topic Modeling

Obesity-related posts were clustered into various topics using BERTopic, with a minimum topic size of 100 [18]. This minimum topic size was chosen to balance the size and similarity of the cluster. The BERTopic modeling process has demonstrated performance improvements over classical topic models, including latent Dirichlet allocation (LDA) and nonnegative matrix factorization when applied to both social media and public health data [19-23]. For example, a recent publication by de Groot and colleagues [23] showed that BERTopic generalizes well to short text domains (such as social media) and outperforms LDA in terms of coherence and diversity of topics. BERTopic first extracts document embeddings using bidirectional encoder representations from transformers (BERT), which generates numerical representations of textual data that preserve the context of the original text [24]. Embeddings then undergo dimensionality reduction before hierarchical clustering methods are applied to categorize them into topics. BERTopic was used independently on Facebook and Instagram data. Topic themes were assigned by a member of the research team with expertise in obesity medicine via qualitative examination of the top 3 exemplar points for each topic (ie, content located in the densest area of each cluster). Topics with exemplar posts that were focused on pet obesity were excluded. Temporal modeling, as described above, was conducted on the finalized set of top 10 topics.

For all analyses, a Bonferroni-adjusted critical value of *P*≤.003 was chosen by dividing the typical *P*≤.05 threshold by 16 (ie, 4 dates across 2 platforms with 2 types of content) to apply a conservative adjustment for multiple comparisons. Topic coherence was evaluated with c_v_ coherence, whereby values closer to 1 represent more intracluster similarity. Analyses were conducted in Python (version 3.8.8; Python Software Foundation) and R (version 4.1.12; R Foundation) using Visual Studio Code (version 1.63.2; Microsoft Corp) and RStudio (version 2021.09.0; Posit Software), respectively.

### Ethical Considerations

Institutional review board approval was not required for this study given the public-facing nature of the social media data used [25].

## Results

### Aggregate Analysis

#### Facebook

Between January 6 and October 16, 2020, there were 175,242 posts across 66,497 public Facebook pages, groups, and pages in the CrowdTangle repository that contained the words “obesity” or “obese.” There was no significant change in posting behavior in the 14 days after January 20 and March 11 compared to the 14 days prior (Table 1). There was a significant pulse (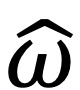
_p,2_) effect on May 19 (ie, the approximate date when COVID-19 and obesity were linked), with a temporary increase of 405 posts (95% CI 166 to 645; *P*<.003). This was not observed in the health control data (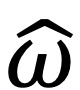
_p,2_=104, 95% CI –63.3 to 271; *P*=.224) or nonhealth control data (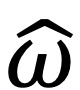
_p,2_=87.4, 95% CI –23.7 to 198; *P*=.123). While the best model for this period also included a step parameter (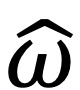
_s,2_) for a sustained effect in the 14 days after the event, this was not significant at the Bonferroni-adjusted threshold (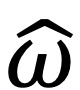
_s,2_=500, 95% CI 60.0 to 941; *P*=.026). The October 2 model included a pulse (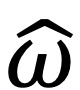
_p,3_) of 639 posts that was statistically significant (95% CI 359 to 883; *P*<.003). This effect was not observed in the health control data (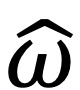
_p,3_=268, 95% CI 87.1 to 450; *P*=.004) or nonhealth control data (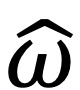
_p,3_=196, 95% CI 27.4 to 364; *P*=.023) at the Bonferroni-adjusted threshold. The ramp parameter in the 14 days after the event in the obesity model was also not significant at the Bonferroni-adjusted threshold (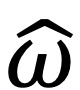
_r,3_=9.06, 95% CI 2.84 to 15.3; *P*=.004).

**Table 1:** Autoregressive integrated moving average models for Facebook posts per day. Values in italics denote statistical significance at the Bonferroni-adjusted threshold of *P*≤.003.

Category/date (parameters)				Estimate (95% CI)		*P* value^a^
**Obesity**						
	**January 20, 2020 (p=0, d=0, q=1, AICc^b^=341.24)**					
		Ramp ( 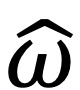 _r,0_)^c^	–3.00 (–12.0 to 6.03)		.515	
	**March 11, 2020 (p=5, d=2, q=0, AICc=367)**					
		Pulse ( 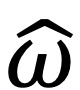 _p,1_)^d^	–126 (–322 to 70.3)		.209	
	**May 19, 2020 (** ***p=0, d=1, q=1, AICc=358.6*** **)**					
		Pulse ( 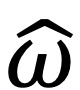 _p,2_)	*405 (166 to 645)*		*<.003*	
		Step ( 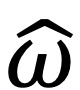 _s,2_)^e^	500 (60.0 to 941)		.026	
	**October 2, 2020 (** ***p=3, d=0, q=0, AICc=382.94*** **)**					
		Pulse ( 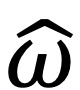 _p,3_)	*639 (395 to 883)*		*<.003*	
		Ramp ( 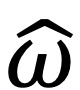 _r,3_)	9.06 (2.84 to 15.3)		.004	
**Health control data**						
	**January 20, 2020 (p=4, d=1, q=0, AICc=356.98)**					
		Ramp ( 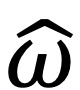 _r,0_)	–0.67 (–29.8 to 28.5)		.964	
	**March 11, 2020 (p=0, d=1, q=0, AICc=360.43)**					
		Pulse ( 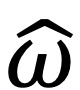 _p,1_)^e^	–14.5 (–208 to 179)		.883	
	**May 19, 2020 (** ***p=5, d=0, q=0, AICc=360.63*** **)**					
		Pulse ( 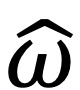 _p,2_)	87.4 (–23.7 to 198)		.123	
		Step ( 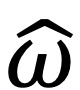 _s,2_)	–*71.8 (–94.8 to –48.8)*		*<.003*	
	**October 2, 2020 (p=4, d=0, q=0, AICc=370.54)**					
		Pulse ( 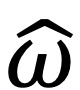 _p,3_)	196 (27.4 to 364)		.023	
		Ramp ( 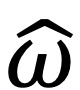 _r,3_)	–6.08 (–10.6 to –1.56)		.008	
**Nonhealth control data**						
	**January 20, 2020 (p=0, d=0, q=0, AICc=287.6)**					
		Ramp ( 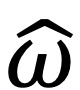 _r,0_)	0.20 (–2.00 to 2.40)		.859	
	**March 11, 2020 (p=1, d=0, q=0, AICc=295.2)**					
		Pulse ( 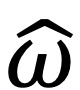 _p,1_)	–29.2 (–93.6 to 35.2)		.374	
	**May 19, 2020 (p=0, d=0, q=1, AICc=299.81)**					
		Pulse ( 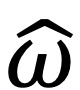 _p,2_)	2.88 (–58.3 to 64.1)		.927	
		Step ( 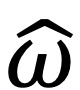 _s,2_)	–17.0 (–54.0 to 20.0)		.368	
	**October 2, 2020 (p=0, d=0, q=0, AICc=298.64)**					
		Pulse ( 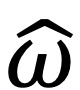 _p,3_)	–25.8 (–96.6 to 45.1)		.476	
		Ramp ( 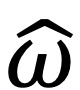 _r,3_)	–0.95 (–3.51 to 1.60)		.464	

^a^*P* values based on an autoregressive integrated moving average model for Facebook posts per day in the specified data set with a Bonferroni-adjusted significance threshold of *P*=.001.

^b^AICc: sample-corrected Akaike information criterion.

^c^Ramp (ω_r_) functions are 0 before the intervention and (t–T+1) after the intervention (where *t* represents the current day and *T* represents the intervention date).

^d^Pulse (ω_p_) functions are 1 if it is the day of the intervention and 0 all other days.

^e^Step (ω_s_) functions are 0 before the intervention and 1 the day of and after the intervention.

Changes in interactions on obesity posts for the 14 days following January 20 and March 11 were not significant (Table 2). On May 19, there were significant pulse (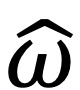
_p,2_=294,930, 95% CI 125,986 to 463,874; *P*<.003) and step (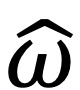
_s,2_=473,247, 95% CI 235,680 to 711,814; *P*<.003) increases in interactions not significant in either control. The included ramp effect (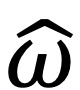
_r,2_) in the obesity model was also not significant at the Bonferroni-adjusted threshold (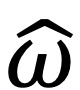
_r,2_=–38,596, 95% CI –64,268 to –12.923; *P*=.003). The October 2 pulse effect (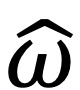
_p,3_) was significant in both the obesity model and health control data, although there were approximately 5000 more interactions on average in the obesity data set (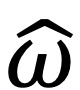
_p,3_=182,814, 95% CI 160,524 to 205,105; *P*<.003) relative to the health control data (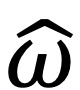
_p,3_=177,855, 95% CI 96,952 to 258,758; *P*<.003). The best model for this date also included a step parameter (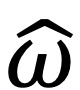
_s,3_), but it was not significant at the Bonferroni-adjusted threshold (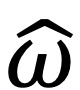
_p,3_=5791, 95% CI 1449 to 10,134; *P*=.009).

**Table 2:** Autoregressive integrated moving average models for Facebook interactions. Values in italics denote statistical significance at the Bonferroni-adjusted threshold of *P*=.003.

Category/date (parameters)				Estimate (95% CI)		*P* value^a^
**Obesity**						
	**January 20, 2020 (p=2, d=1, q=0, AICc^b^=691.73)**					
		Step (ω_s,0_)^c^	–61,169 (–139,294 to 16,957)		.125	
	**March 11, 2020 (p=3, d=1, q=1, AICc=681.75)**					
		Pulse (ω_p,1_)^d^	–3298 (–60,578 to 53,981)		.910	
	**May 19, 2020 (*p=2, d=0, q=0, AICc=762.64*)**					
		Pulse (ω_p,2_)	*294,930 (125,986 to 463,874)*		*<.003*	
		Step (ω_s,2_)	*473,247 (235,680 to 711,814)*		*<.003*	
		Ramp (ω_r,2_)^e^	–38,596 (–64,268 to –12,923)		.003	
	**October 2, 2020 (*p=3, d=0, q=0, AICc=661.31*)**					
		Pulse (ω_p,3_)	*182,814 (160,524 to 205,105)*		*<.003*	
		Step (ω_s,3_)	5791 (1449 to 10,134)		.009	
**Health control data**						
	**January 20, 2020 (p=2, d=1, q=0, AICc=667.46)**					
		Step (ω_s,0_)	37,827 (–9152 to 84,807)		.115	
	**March 11, 2020 (** ***p=0, d=1, q=1, AICc=686.64*** **)**					
		Pulse (ω_p,1_)	*176,502 (93,695 to 259,308)*		*<.003*	
	**May 19, 2020 (p=0, d=0, q=0, AICc=699.9)**					
		Pulse (ω_p,2_)	–10,492 (–86,771 to 65,786)		.787	
		Step (ω_s,2_)	1826 (–43,247 to 46,890)		.937	
		Ramp (ω_r,2_)	–1139 (–5542 to 3265)		.612	
	**October 2, 2020 (** ***p=0, d=0, q=0, AICc=706.39*** **)**					
		Pulse (ω_p,3_)	*177,855 (96,952 to 258,758)*		*<.003*	
		Step (ω_s,3_)	–49.6 (–29,591 to 29,492)		.997	
**Nonhealth control data**						
	**January 20, 2020 (p=2, d=1, q=0, AICc=479.66)**					
		Step (ω_s,0_)	–107 (–1835 to 1621)		.903	
	**March 11, 2020 (p=0, d=0, q=0, AICc=504.12)**					
		Pulse (ω_p,1_)	–2189 (–4738 to 359)		.092	
	**May 19, 2020 (p=1, d=2, q=0, AICc=491.06)**					
		Pulse (ω_p,2_)	–331 (–3632 to 2969)		.844	
		Step (ω_s,2_)	377 (–5442 to 6196)		.899	
		Ramp (ω_r,2_)	–589 (–4759 to 3582)		.782	
	**October 2, 2020 (p=0, d=0, q=0, AICc=484.48)**					
		Pulse (ω_p,3_)	–138 (–1901 to 1626)		.878	
		Step (ω_s,3_)	66.1 (–578 to 710)		.840	

^a^*P* values based on an autoregressive integrated moving average model for Facebook interactions per day in the specified data set with a Bonferroni-adjusted significance threshold of *P=*.001.

^b^AICc: sample-corrected Akaike information criterion.

^c^Step (ω_s_) functions are 0 before the intervention and 1 the day of and after the intervention.

^d^Pulse (ω_p_) functions are 1 if it is the day of the intervention and 0 all other days.

^e^Ramp (ω_r_) functions are 0 before the intervention and (t–T+1) after the intervention (where *t* represents the current day and *T* represents the intervention date).

#### Instagram

Between January 6 and October 16, 2020, there were 18,129 posts across 3170 unique usernames in the CrowdTangle repository containing “obese” or “obesity.” Of the 4 dates, only a ramp effect after January 20 (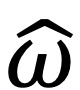
_r,0_) was significant (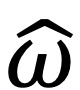
_r,0_=–1.04, 95% CI –1.33 to –0.76; *P*<.003). There was a pulse effect (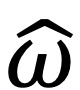
_p,2_) on May 19 of approximately 61 (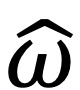
_p,2_=61.1, 95% CI 18.5 to 104; *P*=.005) additional posts, although this was not significant at the Bonferroni-adjusted threshold. For both dates, no significant effect was observed in the health control or nonhealth control data (Multimedia Appendix 1). For interactions, there was a pulse effect (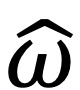
_p,2_) on May 19 of an estimated 226,017 (95% CI 107,323 to 344,708; *P*<.003) additional interactions relative to the surrounding window. This was not observed in either the health control (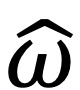
_p,2_=–13,005, 95% CI –218,774 to 192,764; *P*=.880) or nonhealth control (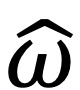
_p,2_=2161, 95% CI –35,026 to 39,349; *P*=.909) data. Similarly, there was a pulse (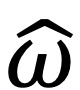
_p,3_) of 156,974 (95% CI 89,757 to 224,192; *P*<.003) additional interactions on obesity content on October 2 not observed at the Bonferroni-adjusted threshold in the health control (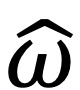
_p,3_=–14,864; 95% CI –246,793 to 217,063; *P*=.900) or nonhealth control (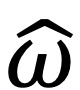
_p,3_=26,307; 95% CI 7774 to 44,840 *P*=.005) data.

### Topic Analysis

#### Facebook—General Description

Of 175,242 obesity-related posts, 87,470 (49.9%) could not be classified into a topic; a random sample of these can be found in Multimedia Appendix 2. The remaining posts were clustered into 245 different topics with a c_v_ topic coherency of 0.76. Of the initial most frequent topics (Multimedia Appendix 2), 2 were removed because they were related to pet obesity, and 1 was removed because it consisted of 1 redundant post on a male supplement not directly related to obesity. The remaining 10 largest topics contained 19,485 posts, representing 22.2% (19,485/87,772) of classifiable posts and 11.1% (19,485/175,242) of all obesity-related posts. Themes included COVID-19 (n=3849), childhood obesity (n=2443), sugary drinks (n=2425), bariatric surgery (n=2413), weight loss stories (n=2090), “clickbait” (ie, catchy content designed to increase clicks; n=1494), cancer (n=1355), sleep (n=1166), yoga (n=1130), and heart disease (n=1120). Posts related to weight loss stories had the highest median interactions (20, IQR 3-94), while posts related to yoga had the fewest (2, IQR 0-9). Of posts from pages with a labeled category, the most frequent categories included general health sites (sugary drinks, weight loss stories, and sleep themes), hospitals (bariatric surgery, cancer, and heart disease themes), media and news companies (COVID-19 and clickbait themes), nonprofit organizations (childhood obesity theme), and yoga and Pilates (yoga theme).

#### Facebook—Temporal Modeling

**Figure 1:**
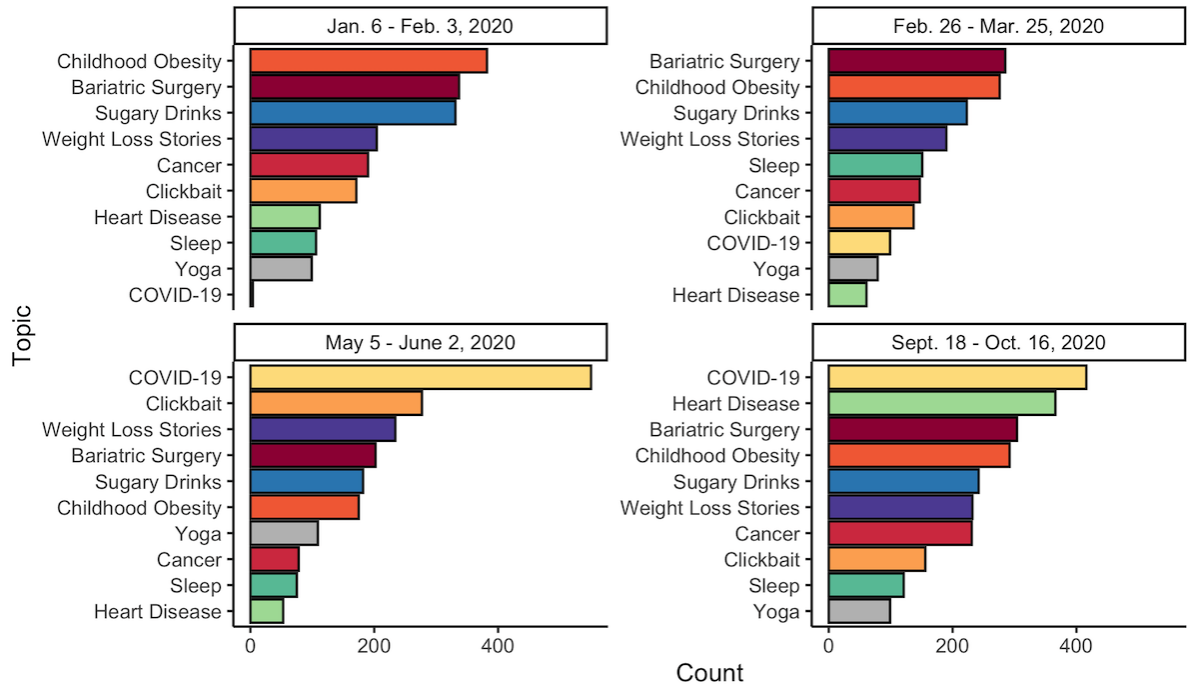
Distribution of the top 10 most frequent topics for Facebook around each date of interest.

The distribution of most frequent topics changed around each date (Figure 1). In the 29 days surrounding and including January 20, the least popular topics were yoga and COVID-19, while the most were childhood obesity and bariatric surgery. These topics remained the most popular around March 11, while the least popular were yoga and heart disease. The distribution changed surrounding May 19, whereby COVID-19 and clickbait were the most popular topics while sleep and heart disease were the least popular. This again changed around October 2, when COVID-19 and heart disease were the most popular topics and sleep and yoga were the least popular.

**Figure 2:**
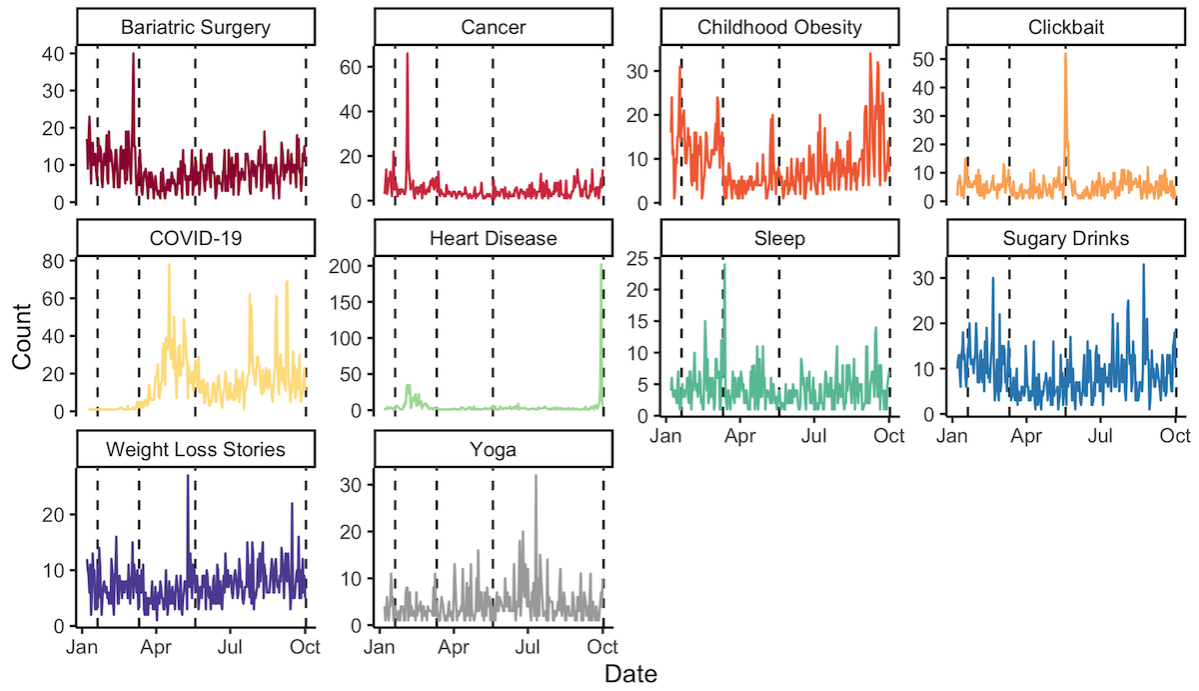
Longitudinal variations in top 10 most frequent topics about obesity on Facebook. The dashed lines indicate the 4 key dates of interest (January 20, March 11, May 19, and October 2, 2020).

Each topic also had distinct daily posting behavior (Figure 2, Multimedia Appendix 3). While there was no significant difference for any topic around January 20, 5 topics showed a change in posting behavior around March 11. The COVID-19 topic experienced an average daily increase of approximately one additional post per day (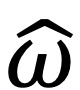
_r,1_=0.69, 95% CI 0.42 to 0.69; *P*<.003). A significant, gradual decline was observed for sugary drinks (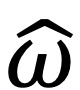
_,1_=–0.48, 95% CI –0.78 to –0.19; *P*<.003) and weight loss stories (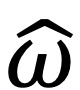
_r,1_=–0.32, 95% CI –0.52 to –0.12; *P*<.003), while an immediate, sustained decline was observed for childhood obesity (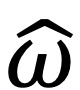
_s,1_=–10.2, 95% CI –12.7 to –7.66; *P*<.003) and clickbait (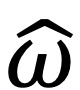
_s,1_=–3.70, 95% CI –5.49 to –1.92; *P*<.003). In contrast, clickbait experienced an immediate pulse of content (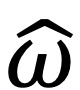
_p,2_=25.2, 95% CI 14.1 to 36.4; *P*<.003) on May 19, coupled with a sustained increase (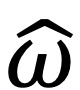
_s,1_=22.1, 95% CI 14.8 to 29.4; *P*<.003) and gradual decrease (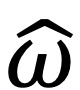
_r,2_=–2.58, 95% CI –3.48 to –1.69; *P*<.003). The cancer topic also experienced a pulse increase (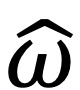
_p,2_=6.34, 95% CI 2.80 to 9.88; *P*<.003), while weight loss stories experienced a step decrease (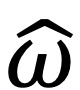
_s,2_=–2.85, 95% CI –4.52 to –1.19; *P*<.003). No topics met the Bonferroni-adjusted statistical significance threshold for October 2, although clickbait experienced a sustained increase of approximately two additional posts per day (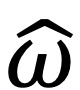
_p,3_=1.95, 95% CI 0.17 to 3.73; *P*=.032).

#### Instagram—General Description

Of the 18,129 obesity-related Instagram posts, 6856 (37.8%) could not be classified into a topic; a random sample of these can be found in Multimedia Appendix 4. The remaining posts were clustered into 28 different topics with a c_v_ topic coherency of 0.57. Of the initial 10 largest topics, 1 (with n=769 posts) was removed for its pet-specific content. The remaining 10 largest topics represented 60.9% (6865/11,273) of classifiable posts and 37.9% (6865/18,129) of all posts (Multimedia Appendix 4). Some themes overlapped with Facebook, including weight loss stories (n=2718 posts), COVID-19 (n=1069 posts), bariatric surgery (n=363 posts), childhood obesity (n=331 posts), and sleep (n=312 posts). Additional topics included keto diet (n=588 posts), specific weight loss programs (n=415 posts), calories (n=391 posts), sugar (n=341 posts), and responses to a UK government policy (n=337 posts). Weight loss stories had the highest median overall interactions (544, IQR 154-1733), while the bariatric surgery topic had the fewest (51, IQR 17-114).

#### Instagram—Temporal Modeling

**Figure 3:**
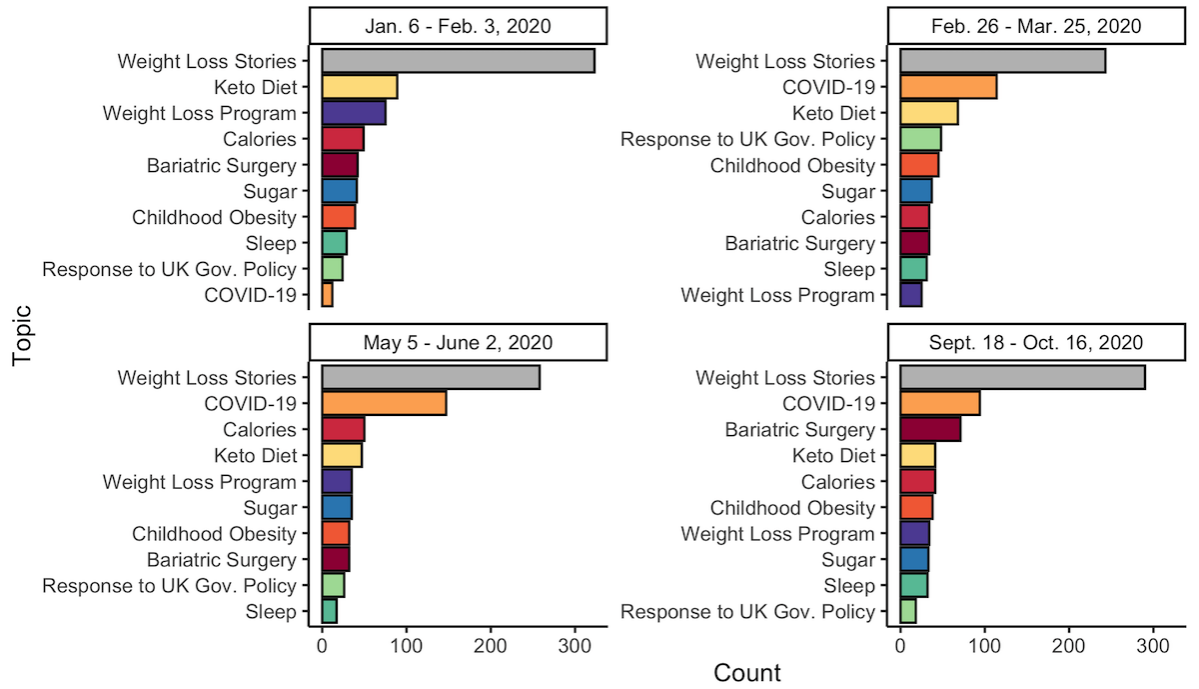
Distribution of the top 10 most frequent topics for Instagram around each date of interest.

The ranking of topic frequency was consistent around each date (Figure 3). Weight loss stories were the most frequent in each of the 4 windows, and COVID-19 was the second most frequent in 3 of the 4. The only exception was the first window, in which COVID-19 was the least frequent and the keto diet was the second most frequent. The least frequent topic varied within the other 3 windows—the weight loss program was the least frequent around March 11, sleep was the least frequent around May 19, and responses to the UK government policy were the least frequent around October 2.

**Figure 4:**
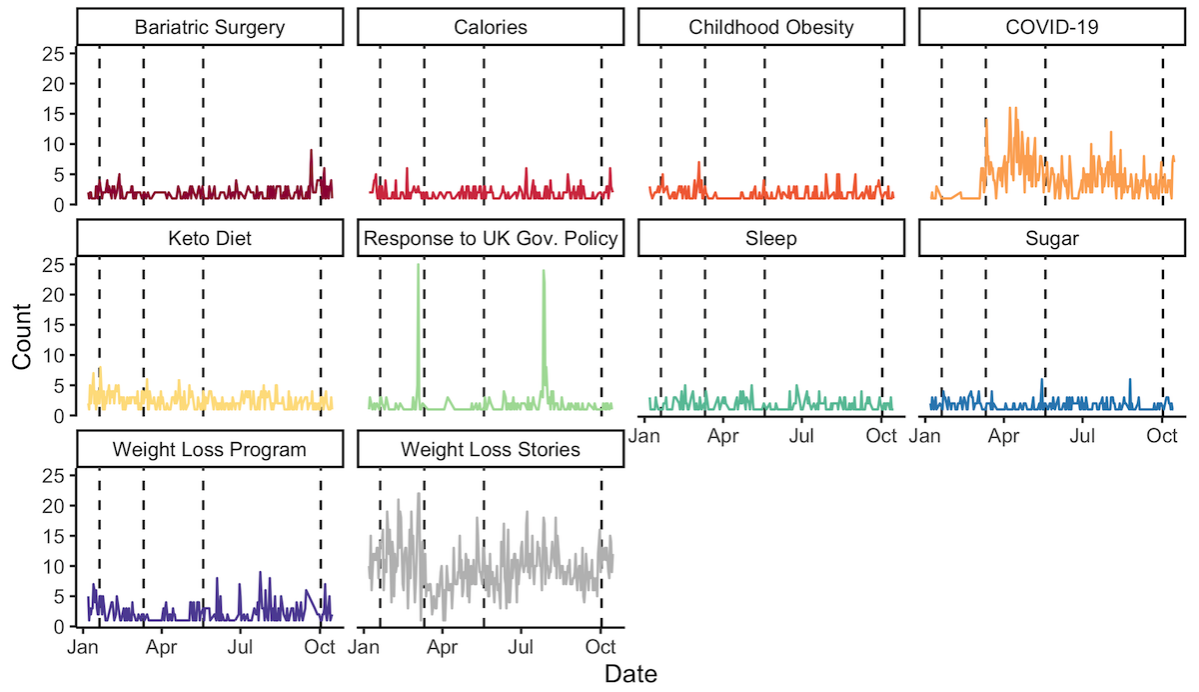
Longitudinal variations in top 10 most frequent topics about obesity on Instagram. The dashed lines indicate the 4 key dates of interest (January 20, March 11, May 19, and October 2, 2020).

Two Instagram topics changed significantly surrounding the 4 dates (Figure 4, Multimedia Appendix 5). On January 20, there was a significant pulse increase (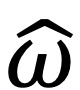
_p,0_) in keto posts (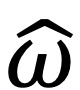
_p,0_=4.88, 95% CI 1.84 to 7.93; *P*<.003), and on October 2, there was a sustained increase (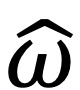
_s,3_) in posts about calories (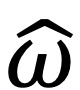
_s,3_=1.36, 95% CI 0.51 to 2.21; *P*<.003). Other topics also showed a change in posting behavior that did not reach the Bonferroni-adjusted significance threshold. Posts on the childhood obesity topic experienced a sustained decrease of 1.17 posts (95% CI –2.22 to –0.11; *P*=.030). This also occurred on March 11, with an immediate, sustained decrease of 1.59 posts (95% CI –3.05 to –0.12; *P*=.034). Weight loss stories experienced a gradual ramp (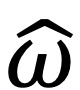
_r,1_) decrease of 0.61 posts (95% CI –1.02 to –0.21; *P*=.003). In the 14 days following May 19, topics related to weight loss stories (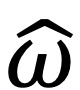
_r,2_=–0.23, 95% CI –0.44 to –0.03; *P*=.026), COVID-19 (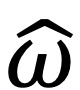
_r,2_=–0.20, 95% CI –0.38 to –0.02; *P*=.028), and calories (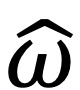
_r,2_=–0.14, 95% CI –0.25 to –0.02; *P*=.022) experienced a gradual ramp decline. Around October 2, topics related to sugar (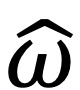
_p,3_=1.50, 95% CI 0.34 to 2.66; *P*=.011) and childhood obesity (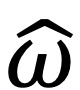
_p,3_=1.83, 95% CI 0.18 to 3.49; *P*=.030) experienced a pulse increase, weight loss stories experienced a step increase (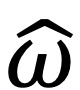
_s,3_=2.43, 95% CI 0.55 to 4.30; *P*=.011), and COVID-19 topics experienced a ramp increase (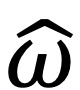
_r,3_=0.18, 95% CI 0.04 to 0.32; *P*=.010).

## Discussion

This study is the first to comprehensively evaluate obesity-related content throughout the pandemic on Facebook and Instagram. On Facebook, obesity-related content surged around the dates of 4 key news stories related to obesity or COVID-19. Posting behavior of obesity-related content on Instagram was not affected, although changes in interactions occurred. Frequent content on each platform had some overlapping themes (ie, COVID-19, bariatric surgery, childhood obesity, weight loss stories, and sleep), while other topics varied in popularity. These findings demonstrate how social media conversations regarding prevalent health conditions may be influenced by news media and global events.

On Facebook, there were immediate changes in posting and interaction behavior for obesity-related content on both May 19 and October 2 that were not sustained in the following 14 days. A similar effect was observed for interactions on Instagram. The lack of statistical significance in the control data for any of the May 19 outcomes provides evidence that change in online discussion about obesity was specific to the reported association between COVID-19 and obesity that was shared in the mainstream media on that day. For October 2, a significant pulse effect was observed for interactions on Facebook posts in the health control data, while the nonhealth control data remained insignificant. Since the keyword for the health control data (ie, “headache”) is also a symptom of COVID-19, this may suggest that the surge in interactions occurred on posts that discussed the same topic as the obesity data (ie, the prognosis of then US president Trump, who had contracted COVID-19).

When broken down by topic, 5 frequent topics overlapped. Three (ie, bariatric surgery, pediatric obesity, and sleep) were clinical in nature and received the fewest interactions from users, suggesting that social media may not be an ideal platform to communicate this kind of content. In contrast, weight loss stories were present on both platforms and received a high number of interactions. This consistency may suggest that individuals feel comfortable sharing personal stories on these platforms as a show of support for others, and frequent mentions of obesity online or in mainstream media may empower individuals to communicate their own experiences with obesity or weight loss [26]. However, anecdotal stories may spread commonly held falsehoods about weight loss or give viewers unrealistic expectations. This is especially problematic on Instagram, which has faced scrutiny over how it may detrimentally impact body image among its adolescent female user base [26,27].

Prior work has pointed to a limited amount of healthy dietary advice on Instagram among posts with the hashtags #weightloss or #quarantine15 [10,11]. The present work adds to that literature, as 3 of the top 10 most frequent themes on Instagram were focused on some kind of diet or food group (ie, keto diet, calories, and sugar). Exemplar posts for each of these categories often included compelling language that promised a lifechanging transformation (in the case of keto) or warned of imminent dangers if immediate changes were not made (in the case of calories and keto). This kind of catchy language was also dominant in the “clickbait” category on Facebook, which consisted primarily of short phrases that encouraged readers to click on either a linked article or shared post. Although the words “obesity” or “obese” were not present in the analyzed text itself, the fact that these posts were included in the data suggests that other information in the posts (such as the image text or link descriptions) included the keywords. Future work is needed to perform an in-depth analysis on this topic to understand the types of links shared and how frequently individuals interacted with them.

The practical implications and importance of these finding are 3-fold. Broadly, the ability to isolate the impact that media mentions of public health topics have on social media discussion contributes to the growing body of literature that demonstrates how social media can help gauge public opinions during times of crisis [27,28]. This study demonstrates that by comparing a public health topic of interest to multiple controls one can obtain quantitative estimates of the effect that major announcements or stories about the disease have on dialogue. While the present study focused on 2 obesity-related events covered by many media outlets, future work could identify stories covered by only a few outlets to try to estimate precise effects of those channels. Additionally, the swift and substantial response across social media platforms to obesity- and COVID-19–related stories in the media emphasizes the need for both researchers and media outlets to consider how premature public health announcements may contribute to the spread of online misinformation. Future work should study whether this strong relationship is present across other health topics, time periods, and platforms. Finally, this study adds to the growing body of literature that demonstrates the utility and power of BERTopic in analyzing both public health and social media data [19-23].

Strengths of this study include its expansion on prior work to understand obesity discourse more broadly, inclusion of multiple social media platforms, and evaluation of both temporal and topical patterns. However, there are several limitations that are important to note. First, there were no demographic data for users who created and viewed the content, which is a common challenge of epidemiologic research on social media. This study attempted to address this in part by using multiple platforms, which broadened the possible generalizability of the study. For instance, while approximately 71% of US adults aged between 18 and 29 years report ever using each platform, only 13% of US adults over the age of 65 years report using Instagram, compared to 50% for Facebook. Differences exist in other demographic groups as well, including race, income, and education [8]. Because each group may vary in how they perceive and discuss obesity (as well as in their underlying risk factors for the disease), future multi-platform studies are of the utmost importance to characterize perceptions of multiple groups. Second, while some content related exclusively to pet obesity was removed during topic analysis, future work could refine this process to ensure that all animal obesity content was removed. Third, only English-language content was evaluated; future work could be expanded to examine content in other languages. Fourth, only a finite number of topics was evaluated; future work could attempt to conduct a more holistic analysis, including exploration of outlier posts that could not be classified or adjustment of the minimum topic size in the BERTopic algorithm. Future work could also explore topics outside of the top 10, as these only represented about 22.2% (19,485/87,772) of classifiable posts about obesity on Facebook. Fifth, the use of a Bonferroni-adjusted threshold resulted in a conservative evaluation of the results, which may have biased findings toward the null (ie, fewer associations were made between the dates of interest and posting or interaction behavior). Finally, this study only looked at 4 dates of interest; future work could evaluate additional dates that occurred either before the pandemic or after 2020.

Overall, these findings suggest that the pandemic had distinct impacts on the frequency of and attention to obesity-related conversations on 2 popular social media platforms. Posts about obesity and corresponding interactions did not shift after two COVID-19–specific dates (ie, January 28 and March 11), suggesting that events of public health significance that do not relate to obesity do not dramatically alter conversations about the disease on Facebook and Instagram. In contrast, posts and interactions about obesity increased after 2 dates of importance to both COVID-19 and obesity (ie, May 19 and October 2). This pattern was not observed in health and nonhealth control data for the same time period, demonstrating how the relationship between COVID-19 and obesity amplified discussions about obesity. Clinical topics were similar between the platforms, as were weight loss stories. Dietary topics were more prevalent on Instagram, while “clickbait” was more prevalent on Facebook. Taken together, these results suggest that the impact of major public health events (including mainstream media attention and government campaigns) on social media discourse can be successfully isolated and monitored. Public health officials should consider leveraging social media campaigns to prevent the spread of misleading, deleterious content, such as misinformation that may spike around such events.

## Appendix

Multimedia Appendix 1 Models with matched p and q parameters.

Multimedia Appendix 2 Representative documents from Facebook topics.

Multimedia Appendix 3 Model selection and equations for Facebook topics.

Multimedia Appendix 4 Representative documents from Instagram topics.

Multimedia Appendix 5 Model selection and equations for Instagram topics.

## Abbreviations

AICc: sample-corrected Akaike information criterion
ARIMA: autoregressive integrated moving average
BERT: bidirectional encoder representations from transformers
LDA: latent Dirichlet allocation
WHO: World Health Organization
