# Supplementary material for: Obesity-Related Discourse on Facebook and Instagram Throughout the COVID-19 Pandemic: Comparative Longitudinal Evaluation
Source: JMIR Infodemiology. 2023 May 16;3:e40005. doi: 10.2196/40005 (PMC10203886; doi:10.2196/40005)
Supplement: Multimedia Appendix 1 [file infodemiology_v3i1e40005_app1.docx]

**Supplementary Materials 1: Models with matched p & q parameters**

| **AutoRegressive Integrated Moving Average (ARIMA) models – Facebook Posts Per Day** | | | | | | | | | | |
| --- | --- | --- | --- | --- | --- | --- | --- | --- | --- | --- |
| **Time Point** | **Estimate (95% CI)**  **Obesity**  **p,d,q (AIC)** | ***P*-Value** | **Estimate (95% CI)**  **Health Control**  **p,d,q (AIC)**  **Matched p,q** | ***P*-Value** | **Estimate (95% CI)**  **Health Control**  **p,d,q (AIC)**  **Not Matched** | ***P*-Value** | **Estimate (95% CI)**  **Non-Health Control**  **p,d,q (AIC)**  **Matched p,q** | ***P*-Value** | **Estimate (95% CI)**  **Non-Health Control**  **p,d,q (AIC)**  **Not Matched** | ***P*-Value** |
| January 20^th^  Ramp (ω_r,0_)^e^ | 0,0,1 (341.24)  -3.00 (-12.0, 6.03) | .515 | 0,1,1 (358.73)  6.24 (-75.9, 88.3) | .882 | 4,1,0 (356.98)  -0.67 (-29.8, 28.5) | .964 | 0,0,1 (289.22)  0.33 (-2.19, 2.86) | .796 | 0,0,0 (287.6)  0.20 (-2.00, 2.40) | .859 |
| March 11^th^  Pulse (ω_p,1_)^f^ | 5,2,0 (367)  -126 (-322, 70.3) | .209 | 5,1,0 (368.67)  -40.6 (-274, 193) | .733 | 0,1,0 (360.43)  -14.5 (-208, 179) | .883 | 5,0,0 (302.49)  -81.2 (-138, -24.9) | .005 | 1,0,0 (295.2)  -29.2 (-93.6, 35.2) | .374 |
| **May 19^th^**  **Pulse (ω_p,2_)**  Step (ω_s,2_)^g^ | **0,1,1 (358.6)**  **405 (166, 645**)  500 (60.0, 941) | **< .003**  .026 | 0,0,1 (364.94)  104 (-63.3, 271)  -80.8 (-63.3, 270) | .224  .206 | **5,0,0 (360.63)**  87.4 (-23.7, 198)  **-71.8 (-94.8, -48.8)** | .123  **< .003** | 0,0,1 (299.81)  2.88 (-58.3, 64.1)  -17.0 (-54.0, 20.0) | .927  .368 | 0,0,1 (299.81)  2.88 (-58.3, 64.1)  -17.0 (-54.0, 20.0) | .927  .368 |
| **October 2^nd^**  **Pulse (ω_p,3_)**  Ramp (ω_r,3_) | **3,0,0 (382.94)**  **639 (395, 883)**  9.06 (2.84, 15.3) | **< .003**  .004 | 3,0,0 (372.1)  268 (87.1, 450)  -5.45 (-12.7, 1.79) | .004  .140 | 4,0,0 (370.54)  196 (27.4, 364)  -6.08 (-10.6, -1.56) | .023  .008 | 3,0,0, (305.6)  -33.4 (-98.6, 32.1)  -1.00 (-3.62, 1.61) | .318  .452 | 0,0,0 (298.64)  -25.8 (-96.6, 45.1)  -0.95 (-3.51, 1.60) | .476  .464 |

| **Interactions Per Day - Facebook** | | | | | | | | | | |
| --- | --- | --- | --- | --- | --- | --- | --- | --- | --- | --- |
| **Time Point** | **Estimate (95% CI)**  **Obesity**  **p,d,q (AIC)** | ***P*-Value** | **Estimate (95% CI)**  **Health Control**  **p,d,q (AIC)**  **Matched p,q** | ***P*-Value** | **Estimate (95% CI)**  **Health Control**  **p,d,q (AIC)**  **Not Matched** | ***P*-Value** | **Estimate (95% CI)**  **Non-Health Control**  **p,d,q (AIC)**  **Matched p,q** | ***P*-Value** | **Estimate (95% CI)**  **Non-Health Control**  **p,d,q (AIC)**  **Not Matched** | ***P*-Value** |
| January 20^th^  Step | 2,1,0 (691.73)  -61,169 (-139,294; 16,957) | .125 | 2,1,0 (667.46)  37,827 (-9,152; 84,807) | .115 | 2,1,0 (667.46)  37,827 (-9,152; 84,807) | .115 | 2,1,0 (479.66)  -107 (-1,835; 1,621) | .903 | 2,1,0 (479.66)  -107 (-1,835; 1,621) | .903 |
| March 11^th^  Pulse | 3,1,1 (681.75)  -3,298 (-60,578; 53,981) | .910 | **3,1,1 (694.5)**  **189,528 (99,626; 279,429)** | **< .003** | **0,1,1 (686.64)**  **176,502 (93,695; 259,308)** | **< .003** | 3,0,1 (514.86)  -1,253 (-3,933; 1,428) | .360 | 0,0,0 (504.12)  -2,189 (-4,738; 359) | .092 |
| **May 19^th^**  **Pulse**  **Step**  Ramp | **2,0,0 (762.64)**  **294,930 (125,986; 463,874)**  **473,247 (235,680; 711,814)**  -38,596 (-64,268; -12,923) | **< .003**  **< .003**  .003 | 2,0,0 (706.27)  -10,796 (-86,446; 64,854)  1,839 (-41,519; 45,197)  -1,128 (-5,386; 3,129) | .779  .934  .603 | 0,0,0 (699.9)  -10,492 (-86,771; 65,786)  1,826 (-43,247; 46,890)  -1,139 (-5,542; 3,265) | .787  .937  .612 | 2,2,0 (493.96)  -700 (-4,221; 2,820)  1,076 (-4,820; 6,972)  -342 (-4,089; 3,406) | .697  .721  .858 | 1,2,0 (491.06)  -331 (-3,632; 2,969)  377 (-5,442; 6,196)  -589 (-4,759; 3,582) | .844  .899  .782 |
| **October 2^nd^**  **Pulse**  Step | **3,0,0 (661.31)**  **182,814 (160,524; 205,105)**  5,791 (1,449; 10,134) | **< .003**  .009 | **3,0,0 (715.56)**  **169,562 (70,958; 268,167)**  458 (-35,932; 36,849) | **< .003**  .980 | **0,0,0 (706.39)**  **177,855 (96,952; 258,758)**  -49.6 (-29,591; 29,492) | **< .003**  .997 | 3,0,0 (492.18)  100.6 (-1,573; 1,774)  104 (-435; 644) | .906  .704 | 0,0,0 (484.48)  -138 (-1,901; 1,626)  66.1 (-578; 710) | .878  .840 |

| **Posts Per Day - Instagram** | | | | | | | | | | |
| --- | --- | --- | --- | --- | --- | --- | --- | --- | --- | --- |
| **Time Point** | **Estimate (95% CI)**  **Obesity**  **p,d,q (AIC)** | ***P*-Value** | **Estimate (95% CI)**  **Health Control**  **p,d,q (AIC)**  **Matched p,q** | ***P*-Value** | **Estimate (95% CI)**  **Health Control**  **p,d,q (AIC)**  **Not Matched** | ***P*-Value** | **Estimate (95% CI)**  **Non-Health Control**  **p,d,q (AIC)**  **Matched p,q** | ***P*-Value** | **Estimate (95% CI)**  **Non-Health Control**  **p,d,q (AIC)**  **Not Matched** | ***P*-Value** |
| **January 20^th^**  **Ramp** | **5,0,0 (239.09)**  **-1.04 (-1.33, -0.76)** | **< .003** | **5,0,0 (247.2)**  **-1.36 (-1.99, -0.72)** | **< .003** | 0,0,0 (240.11)  -1.25 (-2.22, -0.28) | .011 | **5,0,0 (181.19)**  **0.27 (0.15, 0.39)** | **< .003** | 0,0,0 (177.11)  0.23 (-0.10, 0.55) | .174 |
| March 11^th^  Step | 4,3,2 (267.34)  7.74 (-38.0, 53.5) | .740 | **4,0,2 (246.28)**  **8.10 (6.37, 9.83)** | **< .003** | **5,0,1 (237.86)**  **8.17 (6.60, 9.73)** | **< .003** | **4,0,2 (200.95)**  **4.46 (2.65, 6.28)** | **< .003** | 0,0,0 (185.83)  4.36 (0.51, 8.21) | .026 |
| May 19^th^  Pulse | 5,1,0 (237.54)  61.1 (18.5, 104) | .005 | 5,0,0 (261.61)  6.25 (-22.4, 34.9) | .670 | 0,1,1 (236.96)  4.66 (-21.4, 30.7) | .726 | 5,0,0 (197.28)  3.47 (-5.09, 12.0) | .427 | 0,0,1 (187.07)  6.17 (-4.13, 16.5) | .240 |
| October 2^nd^  Pulse | 0,0,1 (258.43)  33.2 (1.25, 65.1) | .042 | 0,0,1 (249.19)  19.1 (-11.5, 49.7) | .221 | 4,0,0 (248.79)  23.4 (0.43, 46.4) | .046 | 0,0,1 (193.47)  16.3 (4.84, 27.7) | .005 | 0,0,0 (190.89)  16.4 (4.89, 27.9) | .005 |

| **Interactions Per Day - Instagram** | | | | | | | | | | |
| --- | --- | --- | --- | --- | --- | --- | --- | --- | --- | --- |
| **Time Point** | **Estimate (95% CI)**  **Obesity**  **p,d,q (AIC)** | ***P*-Value** | **Estimate (95% CI)**  **Health Control**  **p,d,q (AIC)**  **Matched p,q** | ***P*-Value** | **Estimate (95% CI)**  **Health Control**  **p,d,q (AIC)**  **Not Matched** | ***P*-Value** | **Estimate (95% CI)**  **Non-Health Control**  **p,d,q (AIC)**  **Matched p,q** | ***P*-Value** | **Estimate (95% CI)**  **Non-Health Control**  **p,d,q (AIC)**  **Not Matched** | ***P*-Value** |
| January 20^th^  Step | 1,0,0 (699.95)  -17,719 (-38,185; 2,748) | .090 | 1,0,0 (784.36)  -18,130 (-117,767; 81,506) | .721 | 0,0,0 (781.9)  -9,488 (-121,358; 102,382) | .868 | 1,0,0 (650.42)  -11,771 (-22,841; -702) | .037 | 0,0,0 (647.71)  -11,758 (-22,823; -693) | .037 |
| March 11^th^  Ramp | 0,0,1 (696.46)  -2,952 (-6,063, 160) | .063 | 0,0,1 (724)  3,732 (-388, 7853) | .076 | 0,0,0 (721.33)  3,791 (-94.0, 7,677) | .056 | **0,0,1 (654.05)**  **1,808 (679, 2,937)** | **.002** | **0,0,0 (651.37)**  **1,809 (646, 2,972)** | **.002** |
| **May 19^th^**  **Pulse** | **0,0,0 (726.9)**  **226,017 (107,323; 344,708)** | **< .003** | 0,0,0 (758.81)  -13,005 (-218,774; 192,764) | .901 | 0,0,1 (757.78)  13,500 (-162,467; 189,468) | .880 | 0,0,0 (659.59)  2,161 (-35,027; 39,349) | .909 | 0,0,0 (659.59)  2,161 (-35,026; 39,349) | .909 |
| **October 2^nd^**  **Pulse** | **0,0,0 (693.92)**  **156,974 (89,757; 224,192)** | **< .003** | 0,0,0 (767.75)  -14,864 (-246,793; 217,063) | .900 | 0,0,0 (767.75)  -14,864 (-246,793; 217,063) | .900 | 0,0,0 (633.34)  25,563 (1,789; 49,338) | .035 | 0,0,1 (628.61)  26,307 (7,774; 44,840) | .005 |
