# Supplementary material for: Obesity-Related Discourse on Facebook and Instagram Throughout the COVID-19 Pandemic: Comparative Longitudinal Evaluation
Source: JMIR Infodemiology. 2023 May 16;3:e40005. doi: 10.2196/40005 (PMC10203886; doi:10.2196/40005)
Supplement: Multimedia Appendix 2 [file infodemiology_v3i1e40005_app2.docx]

**Supplementary Materials 2: Representative Documents from Facebook Topics**

Representative quotes are derived from the HDBScan portion of BERtopic and represent exemplar points. There were 87,470 posts (49.9%) that could not be classified into a category. Examples of these are included on page 3 - 4 of this document.

Topics 6 and 9 were excluded because they related to pet obesity.

Topic 5 was excluded because it was a redundant post for a dietary supplement not related to obesity.

| Topic  Number | Number of  Posts | Topic “Name” | Representative Quote |
| --- | --- | --- | --- |
| 0 | 3,849 | COVID-19 | “Check out the latest research on "Obesity is Associated with Worst Outcomes in COVID-19: An Analysis of Early Data from New York City" — Hajifathalian et. al. Read more in #ObesityJournal. #TOSCOVID-19….” |
| 1 | 2,443 | Childhood Obesity | “Childhood obesity exists and it's actually pretty scary. As parents, we of course want the best for our children. Here's how to re-gain control over your child's diet.” |
| 2 | 2,425 | Sugary Drinks | “Sugary drinks may increase the risk of obesity and heart disease. Celebrate healthy and tasty alternatives by making them at home. Get tips and tricks:…” |
| 3 | 2,413 | Bariatric Surgery | “Bariatric Surgery is a lot more than a weight loss surgery. It can help to treat many other diseases associated to obesity. Such as, Diabetes, hypertension, breathlessness, infertility, heart risk and many more. Speak to our experts today to know more about this life saving surgery. Call us…” |
| 4 | 2,090 | Weight Loss Stories | “Although most of the lines, veins , and shreds are gone (FOR NOW.... bulk season is in full effect).... I’m happy with where I’m at. I’m sitting at 18.4 % body fat which is lean (.4 % from ultra lean) is a good place to be right now. Bulking season is a mental game and it’s hard watching the scale go up but we do it to come back bigger and better. I didn’t wake up like this and yes I used to be obese and 1 prescription away from high blood pressure and diabetic! Trust the process and listen to your coach…” |
| 5 | 1,807 | XPowerman (removed) | “…#CAUSES 1•Stress & Anxiety. 2•Alcohol and Drugs 3•Diabetes. 4•High blood pressure/hypertension 5•Prostate surgery •Masturbation. 6•Depression. 7•prostate cancer & Baldness ,8•Ulcers & use of chemicals. 9•Low self-esteem. 10•Smoking & Narcotics 11•Hormone imbalance 12•Multiple sclerosis 13•Nerve Damage 14•Atherosclerosis 15•Gloom & Fear 16•Uncertainties, Sadness. 17•Fatigue and Obesity.” |
| 6 | 1,668 | Pet Obesity (removed) | Everybody meet Ben... Big Ben is a whopping 42kgs, more than double the weight he should be. At 10 years old Ben's joints and heart will be under massive strain carrying all that weight. We've started a weight loss plan of a strict diet and minimal gentle exercise just now to try to limit the strain on his body and hopefully catch diabetes and other obesity related health issues. Join us for his fortnightly Friday weigh-ins and to cheer Ben on. #rescuedog #adoptdontshop #olddogsrule #chubby #doggydiet #healthydog #happydog @ PADS |
| 7 | 1,494 | Clickbait | “This is something I've never really thought about but it is very true!” |
| 8 | 1,355 | Cancer | “Cigarette smoking is the number one risk factor for lung cancer. In the United States, cigarette smoking is linked to about 80% to 90% of lung cancer deaths. Using other tobacco products such as cigars or pipes also increases the risk for lung cancer. Tobacco smoke is a toxic mix of more than 7,000 chemicals. . know more:https://www.cancercarepune.com/ #BestCancerSpecialistInPune #Cervicalcancer #OncologistInPune #CancerTreatmentInPune #CancerHospitalsInPune #cancercare #cancerpreventiontips #cancersurvivor #cancerawareness #cancerfighter #cancerwarrior #cancersupport #cancerprevention #cancercure #cancercouncil #cancertreatment #obesity #pharyngeal #exercise #oralcancer” |
| 9 | 1,225 | Pet Obesity (removed) | “Its Pet Obesity Awareness Day!! If your pet is getting a little heavy, give us a call to set up a time to talk with the Doctor. As much as we love to spoil our little furry ones, too much weight on them can lead to all kinds of complications down the road.” |
| 10 | 1,166 | Sleep | “…. According to the Centers for Disease Control and Prevention (CDC), insufficient sleep increases the risk of type 2 diabetes, cardiovascular disease, and obesity….” |
| 11 | 1,130 | Yoga | “🤸‍♀ Free Yoga Sessions🧘‍♀️🙏🏻 Conducting practice sessions daily in *ADI ashtanga YOGA* Youtube channel via Live streaming From *5:45 Am to 6:45 Am* . We will be discussing the different health issues like Sugar, thyroid, joint pains, backpain, obesity,etc and the related yoga practices ( Warmups,suryanamaskaras, Asanas, Pranayamas, Therapy Techniques, Relaxation techniques ). lifestyle tips and food tips to cure each of them….” |
| 12 | 1,120 | Heart Disease | “The most significant risk factor for developing or dying from coronary heart disease for women versus men is: a. obesity b. diabetes c. elevated CRP levels d. high levels of HDL-C Source: Mosby's Comprehensive Review for NCLEX-RN, 18th edition. good luck!😅” |


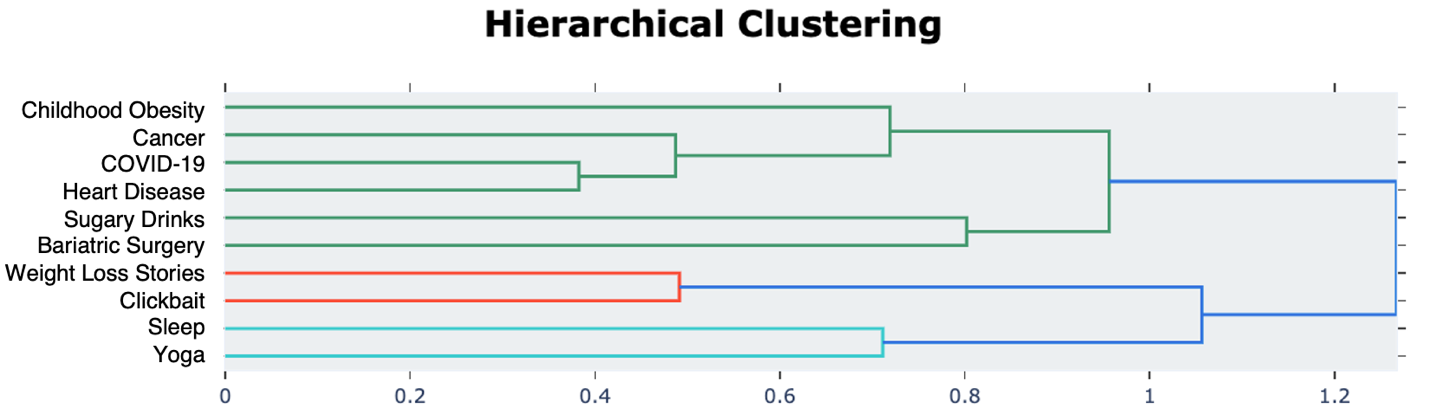


Examples of posts that could not be classified based on a random sample of all unclassifiable posts:

"CDC says 90% of people hospitalized for COVID 19 have underlying conditions. See more here: https://www.usatoday.com/in-depth/news/2020/04/15/coronavirus-risk-90-patients-had-underlying-conditions/2962721001/"

"Adams Memorial Hospital Offers Diabetes Prevention Program (DPP) Classes Start April 30th, 2020 Did you know…...that over 70% of Americans are OVERWEIGHT or Obese and that being OVERWEIGHT is associated with more than 60 health conditions, including Diabetes? Are you OVERWEIGHT and Looking for a FUN way to Shed those Extra Pounds? Make a Change for LIFE in 2020, and join the DPP! You may not realize you have it, but 1 in 3 American’s has Prediabetes or an elevated blood sugar level that is higher than normal but not YET high enough to be diagnosed with DIABETES. If your A1c is 5.7-6.4% or your Fasting Blood Glucose is 100-125mg/dl this program may be for you! A lifestyle change program, like the DPP (Diabetes Prevention Program) at Adams Memorial Hospital, can help you to MANAGE your weight, LEARN how to eat healthier, and GET active! Weekly classes start April 30, 2020, and class size is limited. Select day session from 11:30 am-12:30 pm or night session from 4:30 pm to 5:30 pm. Classes are held in the Strickler Cancer Institute in the Strickler Library (on Hospital Campus). Make a CHANGE FOR LIFE IN 2020, and call Lisa @ (260)724-2145 ext. 11107 to sign-up."

"A woman goes in to see the psychiatrist about her low self-esteem. She is unhealthy, pale and obese. After tearfully explaining her predicament, the doc says, 'Hmm, yes, could you please lie down on the floor under the window? Now over next to the door? Now under the bookshelves? Thank you. He then occupies himself with writing. The patient, exasperated, interrupts him and asks if he has anything he can offer her. 'No,' he says, 'you need to see your specialist in internal diseases about your poor health. 'Then, what was all that stuff you had me do, lying on the floor?' 'Oh, I am having a new white sofa delivered next week and was wondering where to put it.' 😝😂🤣"
