# Supplementary material for: Obesity-Related Discourse on Facebook and Instagram Throughout the COVID-19 Pandemic: Comparative Longitudinal Evaluation
Source: JMIR Infodemiology. 2023 May 16;3:e40005. doi: 10.2196/40005 (PMC10203886; doi:10.2196/40005)
Supplement: Multimedia Appendix 3 [file infodemiology_v3i1e40005_app3.docx]

**Supplementary Materials 3: Model Selection and Equations for Facebook Topics**

|  | **January 20^th^** | | **March 11^th^** | | **May 19^th^** | | **October 2^nd^** | |  |
| --- | --- | --- | --- | --- | --- | --- | --- | --- | --- |
| **Topic Name** | **Best ω**  **p,d,q (AICc)** | **Estimate (95% CI)** | **Best ω**  **p,d,q (AICc)** | **Estimate (95% CI)** | **Best ω**  **p,d,q (AICc)** | **Estimate (95% CI)** | **Best ω**  **p,d,q (AICc)** | **Estimate (95% CI)** |  |
| **COVID-19** | NA | NA | **Ramp**  **0,0,0 (144.08)** | **0.69 (0.42, 0.96)*** | Pulse  1,1,0 (202.42) | 9.77 (-3.35, 22.9) | Pulse  0,0,0 (181.79) | 10.5 (-0.43, 21.5) |  |
| **Childhood Obesity** | Ramp  0,0,1 (185.13) | -0.39 (-1.00, 0.23) | **Step**  **0,0,0 (161.32)** | **-10.2 (-12.7, -7.66)*** | Step  0,0,0 (170.40) | -3.66 (-6.61, -0.71)* | Step  2,0,0 (196.21) | -6.43 (-11.8, -1.03)* |  |
| **Sugary Drinks** | Ramp  0,0,1 (157.05) | -0.10 (-0.47, 0.27) | **Ramp**  **0,0,0 (171.60)** | **-0.48 (-0.78, -0.19)*** | Step  0,0,0 (162.26) | 1.50 (-1.06, 4.06) | Ramp  0,0,1 (155.43) | -0.34 (-0.70, 0.02) |  |
| Bariatric Surgery | Pulse  0,0,0 (172.98) | 5.15 (-4.21, 14.5) | Pulse  0,1,0 (195.01) | 0 (-10.1, 10.1) | Pulse  0,0,0 (156.64) | 4.18 (-2.20, 10.6) | Ramp  0,0,0 (172.86) | 0.28 (-0.09, 0.65) |  |
| **Weight Loss Stories** | Ramp  0,0,0 (149.64) | -0.21 (-0.45, 0.04) | **Ramp**  **0,0,0 (148.57)** | **-0.32 (-0.52, -0.12)*** | **Step**  **0,0,1 (169.32)** | **-2.85 (-4.52, -1.19)*** | Ramp  0,0,0 (162.74) | 0.13 (-0.18, 0.44) |  |
| **Clickbait** | Pulse  0,0,0 (148.43) | -1.15 (-7.29, 5.00) | **Step**  **0,0,0 (130.22)** | **-3.70 (-5.49, -1.92)** | **Pulse**  **Step**  **Ramp**  **0,0,0 (168.19)** | **25.2 (14.1, 36.4)***  **22.1 (14.8, 29.4)***  **-2.58 (-3.48, -1.69)*** | Step  1,0,0 (149.40) | 1.95 (0.17, 3.73)* |  |
| **Cancer** | Step  0,0,0 (170.02) | -2.86 (-6.15, 0.44) | Ramp  0,0,0 (134.49) | -0.28 (-0.51, -0.06)* | **Pulse**  **1,0,0 (109.63)** | **6.34 (2.80, 9.88)*** | Ramp  0,0,0 (182.05) | 0.51 (0.08, 0.95)* |  |
| Sleep | Step  0,0,0 (115.30) | 0.57 (-0.67, 1.81) | Step  Ramp  0,0,0 (176.5) | 4.62 (-0.48, 9.72)  -0.60 (-1.10, -0.09)* | Step  0,0,0 (94.81) | -0.76 (-1.69, 0.17) | Pulse  0,0,0 (133.09) | 3.81 (-0.78, 8.40) |  |
| Yoga | Step  Ramp  0,0,0 (132.14) | -3.25 (-5.88, -0.61)*  0.29 (0.01, 0.57)* | Ramp  0,0,0 (119.82) | -0.16 (-0.41, 0.09) | Pulse  1,0,0 (121.98) | -3.55 (-7.07, -0.30)* | Pulse  0,0,0 (132.41) | -1.59 (-6.13, 2.94) |  |
| Heart Disease | Pulse  0,1,0 (154.28) | 0 (-7.59, 7.59) | Pulse  0,1,0 (83.30) | -1.00 (-3.18, 1.18) | Pulse  0,0,0 (85.16) | 2.71 (-0.13, 5.56) | Step  0,0,0 (277.70) | -18.1 (-45.6, 9.31) |  |
| **P* < .05  **Bold** denotes *P* < .003 | | | | | | | | | |
