# Supplementary material for: Obesity-Related Discourse on Facebook and Instagram Throughout the COVID-19 Pandemic: Comparative Longitudinal Evaluation
Source: JMIR Infodemiology. 2023 May 16;3:e40005. doi: 10.2196/40005 (PMC10203886; doi:10.2196/40005)
Supplement: Multimedia Appendix 4 [file infodemiology_v3i1e40005_app4.docx]

**Supplementary Materials 4: Representative Documents from Instagram Topics**

Representative quotes are derived from the HDBScan portion of BERtopic and represent exemplar points. There were 6,856 posts (37.8%) that could not be classified into a category. Examples of these are included on page 3 - 4 of this document.

Topic 2 was excluded from further analysis because it was focused on pet obesity.

| Topic  Number | Number of  Posts | Topic “Name” | Representative Quote |
| --- | --- | --- | --- |
| 0 | 2,718 | Weight Loss Stories | “And this is only the beginning..... .  . .  .  #weightloss #weightlossjourney #extremeweightloss #fitnessjourney #caloriecounting #facetofacefriday #weightlossgoals #weightlosstransformation #healthjourney #myfitnesspal #fat2fit #fitnessgoals #loosingweight #obesity…” |
| 1 | 1,069 | COVID-19 | “According to the CDC, being obese increases your risk of severe illness from #COVID19….” |
| 2 | 769 | Pet Obesity (removed) | “Pudgy Pets? Prevention is key!  We all want to keep our pets at a healthy weight. When our pets become obese this can lead to health concerns and expensive veterinary bills.  Check with your veterinarian to make sure that your pet is a good weight and if it turns out that your pet could lose a few pounds, enlist his/her help….” |
| 3 | 588 | Keto Diet | “3  🛑STOP blaming meat, eggs, dairy for the obesity epidemic🛑 🥩TOTAL CALORIES per day from meat, poultry, fish, seafood, eggs, dairy, nuts, fruits & vegetables:  1970: 971  1990: 995  2010: 975 ❗️No CHANGE❗️ What has increased?🤔 ☠️Added Sugar/Fat  ☠️Processed Carbs/Fat  #keto #lchfdiet #ketogenicdiet #jerf #keto #ketodiet #atkins #atkinsdiet #ancestral #primal #protein #lowcarb #lchf #intermittentfasting #fasting #fastingforweightloss” |
| 4 | 415 | Weight Loss Program | “If you in Atlanta and you a big girl but you ain’t trying to get in my door, you just like being fat‼️‼️ BIG GIRLZ MOVE with Ted‼️‼️ #weightlossjourney #obesity” |
| 5 | 391 | Calories | “Inb4 I’m posting about politics! 😂😂😂 But seriously, if you you feel like you’re eating very few calories and you’re not actually losing weight then something is off. This is for those who have obesity, not for those who are physique competitors who are already super lean….” |
| 6 | 363 | Bariatric Surgery | “Katrina Weaver, M.D., assistant professor of surgery at the University of South Alabama College of Medicine, recently completed advanced training in pediatric/adolescent bariatric surgery and is now offering new surgery options for young patients dealing with obesity….” |
| 7 | 341 | Sugar | “Minimize your sugar intake, added sugar is one of the worst ingredients in the modern diet, as large amounts can harm your metabolic health. High sugar intake is linked to numerous ailments, including obesity, type 2 diabetes, heart disease, and many forms of cancer….” |
| 8 | 337 | UK Government Obesity Policy | “…  We are SO incredibly frustrated at this Tory government’s new distraction campaign of attacking and pathologising fat bodies, using them as a scapegoat for governmental failures and social inequality during this pandemic. We are angry at its plans to use strategies steeped in diet culture and fat phobia to improve public health when we know that dieting is statistically ineffective and weight stigma has harmful effects on health. We are exhausted by the refocused (and triggering) attention on calories, BMI, “the obesity epidemic” and fatness in general all over the media. We’re currently working to put together an open letter and MP letter template so that we can let our representatives know WE WILL NOT ACCEPT THIS….” |
| 9 | 331 | Childhood Obesity | “…🆘 33.6% of children and adolescents (5 to 19 years-old) in the 🌎 Americas are overweight and 14.4% are obese, increasing the risk of diabetes, cardiovascular disease and some forms of cancer….” |
| 10 | 312 | Sleep | “…DREAM by Axe Laboratories is a groundbreaking sleep supplement unlike anything we have seen before.  Its cutting edge combination of natural yet powerful botanical compounds is showing results previously only seen in hard, organ damaging pharmaceutical medications.  THE IMPORTANCE OF GOOD SLEEP  Getting a good amount of sleep is incredibly important for your health. Sleep helps your body and brain function properly. A good night’s sleep can improve your learning, memory, decision-making and even your creativity. What’s more, getting sufficient sleep has been linked to a lower risk of heart disease, diabetes, stroke and obesity….” |


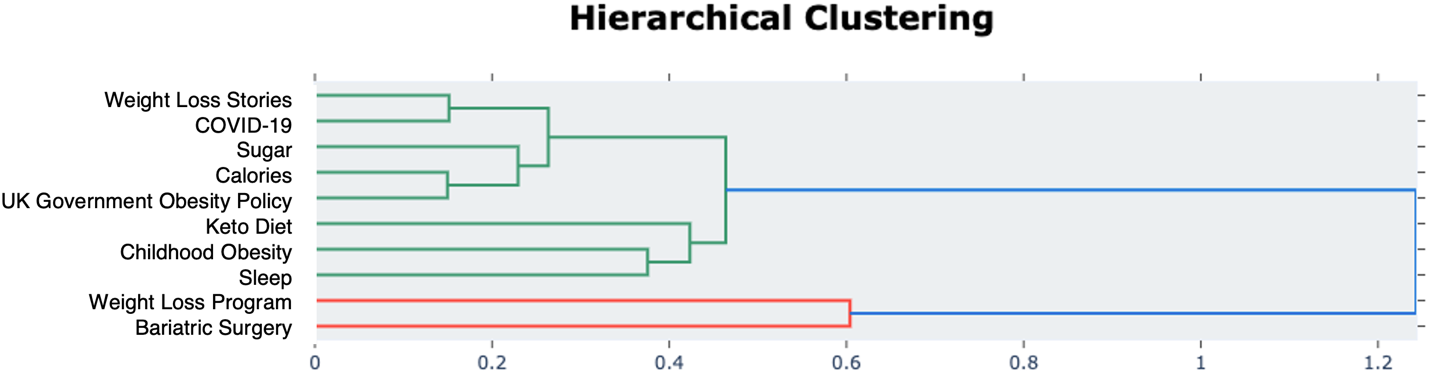


Examples of posts that could not be classified based on a random sample of all unclassifiable posts:

"👉If you keep on eating unhealthy food than no matter how many weight loss tips you follow, you are likely to retain weight and become obese. If only you start eating healthy food, you will be pleasantly surprised how easy it is to lose weight.” \n.\n.\nonline Coach 🏋️\nmail us 📩 kirandembla@gmail.com\n\nmusic🎶👉 lr sais & rauw alejandro (dream girl)\nt shirt @mygalf\n.\n.\n#daughterandmother #daughter #weightloss #partnerworkout"

"😂😂😂 Who knew Spider-Man’s arch nemesis was obesity"

"High blood pressure, obesity, heart disease, diabetes, cardiovascular disease, osteoporosis and cancer...\n.\nDo these sound familiar? They’re all preventable through diet and exercise.\n.\nDNA Fitness educates, equips, and empowers people to build habits that improve their health, increase energy and ultimately transform the quality of their life!\n.\nBook your free intro session today!\n🧡 @DNAfitnessNation 🧡\n🧡 @DNAfitnessNation 🧡"
