# Supplementary material for: Obesity-Related Discourse on Facebook and Instagram Throughout the COVID-19 Pandemic: Comparative Longitudinal Evaluation
Source: JMIR Infodemiology. 2023 May 16;3:e40005. doi: 10.2196/40005 (PMC10203886; doi:10.2196/40005)
Supplement: Multimedia Appendix 5 [file infodemiology_v3i1e40005_app5.docx]

**Supplementary Materials 5: Model Selection and Equations for Instagram Topics**

Note that topics about the weight loss program, sleep, and fruits and vegetables had too few daily posts to be considered

|  | **January 20^th^** | | **March 11^th^** | | **May 19^th^** | | | **October 2^nd^** | |
| --- | --- | --- | --- | --- | --- | --- | --- | --- | --- |
| **Topic Name** | **Best ω**  **p,d,q (AICc)** | **Estimate (95% CI)** | **Best ω**  **p,d,q (AICc)** | **Estimate (95% CI)** | **Best ω**  **p,d,q (AICc)** | **Estimate (95% CI)** | **Best ω**  **p,d,q (AICc)** | | **Estimate (95% CI)** |
| Weight Loss Stories | Step  0,0,0 (153.79) | 1.36 (-1.11, 3.82) | Ramp  0,0,1 (161.09) | -0.61 (-1.02, -0.21)* | Ramp  0,0,0 (149.89) | -0.23 (-0.44, -0.03)* | Step  0,0,0 (138.43) | | 2.43 (0.55, 4.30)* |
| COVID-19 | NA |  | Pulse  1,0,0 (129.09) | 1.89 (-2.28, 6.06) | Ramp  0,0,1 (128.59) | -0.20 (-0.38, -0.02)* | Ramp  0,0,0 (117.90) | | 0.18 (0.04, 0.32)* |
| **Keto Diet** | **Pulse**  **0,0,0 (106.39)** | **4.88 (1.84, 7.93)*** | Ramp  0,0,0 (91.82) | -0.04 (-0.15, 0.08) | Step  0,0,0 (71.03) | 0.71 (-0.28, 1.71) | Step  Ramp  0,0,0 (66.48) | | 1.57 (-0.02, 3.17)  -0.32 (-0.66, 0.02) |
| Weight Loss Program | Ramp  0,0,0 (89.49) | -0.19 (-0.53, 0.14) | NA |  | NA |  | NA | |  |
| **Calories** | Step  0,0,0 (78.20) | -0.76 (-1.71, 0.19) | Pulse  2,1,0 (39.44) | 0.88 (-0.02, 1.78) | Step  Ramp  0,0,0 (66.04) | 0.87 (-0.06, 1.81)  -0.14 (-0.25, -0.02)* | **Step**  **0,0,0 (64.18)** | | **1.36 (0.51, 2.21)*** |
| Bariatric Surgery | Step  0,0,0 (63.85) | 0.64 (-0.20, 1.48) | Step  2,0,0 (48.90) | -0.26 (-0.66, 0.15) | Pulse  0,0,0 (55.72) | 0.33 (-1.40, 2.06) | Ramp  0,0,0 (103.59) | | -0.11 (-0.33, 0.11) |
| Sugar | Pulse  0,0,0 (57.12) | 1.00 (-0.68, 2.68) | Ramp  0,0,0 (60.33) | -0.04 (-0.26, 0.17) | Pulse  0,0,0 (70.76) | 0.26 (-2.10, 2.63) | Pulse  0,0,0 (43.94) | | 1.50 (0.34, 2.66)* |
| UK Government Policy | NA |  | NA |  | Ramp  0,0,0 (42.93) | -0.18 (-0.57, 0.20) | NA | |  |
| Childhood Obesity | Step  0,0,0 (50.40) | -1.17 (-2.22, -0.11)* | Step  0,0,0 (75.26) | -1.59 (-3.05, -0.12)* | Ramp  0,0,0 (55.82) | -0.09 (-0.24, 0.05) | Pulse  Step  0,0,0 (59.75) | | 1.83 (0.18, 3.49)*  -0.83 (-1.58, -0.09)* |
| Sleep | Step  1,0,0 (49.80) | -0.82 (-1.71, 0.07) | Step  1,0,0 (61.05) | -0.92 (-2.01, 0.17) | NA |  | Step  0,0,0 (48.88) | | 0.29 (-0.30, 0.87) |
| **P* < .05  **Bold** denotes *P* < .003 | | | | | | | | | |
